# Transcriptome-wide mapping of internal mRNA *N*^7^-methylguanosine in sporulated and unsporulated oocysts of *Eimeria tenella* reveals stage-specific signatures

**DOI:** 10.1186/s13071-024-06580-3

**Published:** 2024-11-27

**Authors:** Qing-Xin Fan, Zi-Rui Wang, Jin-Long Wang, Yu-Xuan Wang, Ze-Dong Zhang, Lin-Mei Yu, Tao Jia, Xing-Quan Zhu, Qing Liu

**Affiliations:** 1 College of Veterinary Medicine, Shanxi Agricultural University, Taigu, 030801 Shanxi People’s Republic of China; 2 The Yunnan Key Laboratory of Veterinary Etiological Biology, Key Laboratory of Veterinary Public Health of Higher Education of Yunnan Province, College of Veterinary Medicine, Yunnan Agricultural University, Kunming, 650201 Yunnan People’s Republic of China

**Keywords:** *Eimeria tenella*, Oocysts, *N*^7^-methylguanosine, Transcriptomics, Methylated RNA immunoprecipitation sequencing

## Abstract

**Background:**

Growing evidence indicates that *N*^7^-methylguanosine (m^7^G) modification plays critical roles in epigenetic regulation. However, no data regarding m^7^G modification are currently available in *Eimeria tenella*, a highly virulent species causing coccidiosis in chickens.

**Methods:**

In the present study, we explore the distribution of internal messenger RNA (mRNA) m^7^G modification in sporulated and unsporulated oocysts of *E. tenella* as well as its potential biological functions during oocyst development using methylated RNA immunoprecipitation sequencing (MeRIP-seq) and mRNA sequencing (mRNA-seq), and the mRNA-seq and MeRIP-seq data were verified by the quantitative reverse transcription polymerase chain reaction (RT–qPCR) and MeRIP–qPCR, respectively.

**Results:**

Our data showed that m^7^G peaks were detected throughout the whole mRNA body, and the coding DNA sequence (CDS) region displayed the most methylation modification. Compared with unsporulated oocysts, 7799 hypermethylated peaks and 1945 hypomethylated peaks were identified in sporulated oocysts. Further combined analysis of differentially methylated genes (DMGs) and differentially expressed genes (DEGs) showed that there was a generally positive correlation between m^7^G modification levels and gene transcript abundance. Unsurprisingly, the mRNA-seq and MeRIP-seq data showed good consistency with the results of the RT–qPCR and MeRIP–qPCR, respectively. Gene Ontology (GO) and pathway enrichment analysis of DEGs with altered m^7^G-methylated peaks were involved in diverse biological functions and pathways, including DNA replication, RNA transport, spliceosome, autophagy-yeast, and cAMP signaling pathway.

**Conclusions:**

Altogether, our findings revealed the potential significance of internal m^7^G modification in *E. tenella* oocysts, providing some directions and clues for later in-depth research.

**Graphical abstract:**

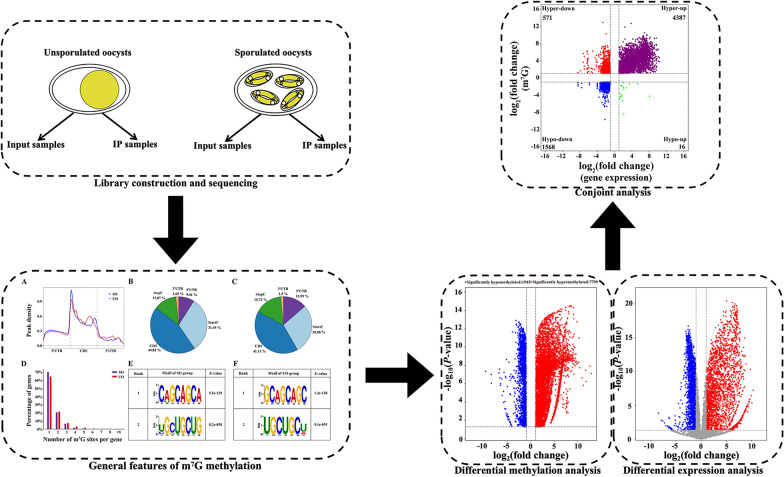

**Supplementary Information:**

The online version contains supplementary material available at 10.1186/s13071-024-06580-3.

## Background

Chicken coccidiosis, an enteric disease caused by apicomplexan parasites belonging to the genus *Eimeria*, is a global problem in the poultry industry [1, 2]. The disease has been estimated to cost about £10.4 billion at 2016 prices in poultry industry [3]. To date, there are ten *Eimeria* species infecting chickens, including seven long-recognized *Eimeria* species and three additional cryptic operational taxonomic units [1, 4]. *Eimeria tenella*, characterized by a unique tropism for the cecum, is among the most economically significant species [5, 6].

*E. tenella* undergoes a strict fecal–oral life cycle, which features an exogenous phase (sporogony) in the environment and an endogenous phase (schizogony and gametogony) within the host [7]. Exogenous and extracellular endogenous life cycle forms include unsporulated oocysts (UO), sporulated oocysts (SO), sporozoites, merozoites, and microgametes [1, 8]. These stages exhibit distinct developmental and morphological characteristics [2]. Several studies aiming to decipher the mechanisms governing the biology of different developmental stages through analysis of the genes expressed as well as the levels of gene expression were performed. For example, a previous study identified 3342 out of 7329 genes exhibiting differential expression during development through comparative transcriptome profiling of *E. tenella* in various developmental stages [9]. The data generated by RNA sequencing (RNA-seq) of *E. tenella* gametocytes, merozoites, and sporozoites revealed upregulated gametocyte transcription of 863 genes [10].

Notably, various RNA modifications are involved in gene expression regulation during eukaryotic development [11, 12]. Over 170 types of RNA modifications have been discovered in eukaryotes thus far, including *N*^6^-methyladenosine (m^6^A), methylcytidine (m^5^C), *N*^1^-methyladenosine (m^1^A) and *N*^7^-methylguanosine (m^7^G) [13]. Of which, m^7^G methylation was initially identified as a signature modification at the 5′ cap of messenger RNAs (mRNAs) [14, 15]. Subsequently, m^7^G methylation was also found at internal positions within mRNAs, ribosomal RNAs (rRNAs), and transfer RNAs (tRNAs) [14]. This modification affects diverse aspects of post-transcriptional gene regulation, including RNA stability and splicing [16]. At the cellular level, the involvement of m^7^G methylation in the processes of differentiation of pluripotent stem cells has been reported [17].

To date, however, the involvement of m^7^G modification in epigenetic regulation during the developmental cycle of *E. tenella* remains unexplored*.* Hence, in the present study, we perform a comprehensive analysis of the transcriptome-wide m^7^G methylation in sporulated and unsporulated oocysts of *E. tenella*.

## Methods

### Chickens and parasites

Sporulated oocysts of *E. tenella* (SD-01 strain) were kindly provided by Professor Xiaomin Zhao, Shandong Agricultural University, China. The strain was maintained by subsequent passage every 6 months as described previously [18]. One-day-old Hy-line Brown cocks, purchased from a commercial hatchery (Shanxi Kangmu Farm, Jinzhong, China), were reared in a coccidia-free environment.

### Preparation of *E. tenella* oocysts

Hy-line Brown cocks were infected orally at 14 days old with a dose of 1 × 10^4^ sporulated oocysts of *E. tenella* SD-01 strain. Seven days postinfection, the chickens were euthanized by cervical dislocation. Fresh oocysts (unsporulated oocysts) were recovered from the cecal contents of infected chickens using procedures described before with minor modifications [19]. The purified unsporulated oocysts were divided into two parts, and each part included three individual aliquots (prepared from different pools of unsporulated oocysts). One part was immediately frozen in liquid nitrogen. The other part was sporulated for 3 days by incubation in 2.5% potassium dichromate solution and then frozen in liquid nitrogen.

### High‑throughput m^7^G sequencing, mRNA sequencing

Total RNA was extracted separately from sporulated and unsporulated oocysts with TRIzol reagent (Invitrogen, CA, USA) according to the manufacturer’s specifications. Then, rRNA was depleted using the GenSeq^®^ rRNA Removal Kit (GenSeq, Inc., Shanghai, China). Regarding fragmentation and immunoprecipitation (IP), the GenSeq^®^ m7G-IP Kit (GS-ET-004, GenSeq Inc., Shanghai, China) was used. For fragmentation, the rRNA depleted samples were treated with fragmentation buffer for 6 min at 70 °C and then halted with stop buffer. Following overnight incubation at −80 °C with PC buffer, PC enhancer, and 75% ethanol, the RNA precipitate was obtained by centrifugation at 15,000*g* for 25 min at 4 °C. After washing by 75% ethanol, the RNA pellet was allowed to air dry at room temperature and dissolved in nuclease-free water. A portion of the resultant RNA samples was kept as input. The remaining was decapped by treatment with tobacco acid pyrophosphatase (TAP) (M0608S, NEB, Ipswitch, MA) at 37 °C for 1 h and then subjected to immunoprecipitation.

For immunoprecipitation, the porcine gastric mucin-conjugated (PGM) magnetic beads were washed with 1× IP buffer and then incubated with the anti-m^7^G antibody (RN017M, MBL, Tokyo, Japan) at room temperature (RT) for 1 h. Following washing with 1× IP buffer, 50 µL of 5× IP buffer, and 250 µL of fragmented RNA were added to the PGM magnetic beads and then incubated at 4 °C for 1 h. The PGM beads were washed with 1× IP buffer, added with 55 µL of dig solution and rotated at 4 °C for 45 min. Afterward, the supernatant was transferred to a new tube with preadded MS beads and RLT buffer. Following incubation with ethanol and washing with 75% ethanol, RNA was eluted with nuclease-free water (immunoprecipitated samples) and used for subsequent library construction with the GenSeq^®^ Low Input RNA Library Prep Kit (GenSeq Inc., Shanghai, China). Meanwhile, RNA input samples without immunoprecipitation were used for RNA library generation with the same kit. Library sequencing was carried out on an Illumina NovaSeq platform at CloudSeq Inc. (Shanghai, China).

### Data analysis

After 3′ adapter trimming and removal of low quality reads by using cutadapt software (v1.18) [20], the clean reads of m^7^G-IP and input libraries were compared with the reference genome of *E. tenella* (ToxoDB-60_EtenellaHoughton2021, https://toxodb.org/toxo/app/downloads/Current_Release/EtenellaHoughton2021/) with the use of HISAT2 software [21]. After mapping, the sequence alignment map (SAM) files were converted to a binary alignment map (BAM) file using SAMtools [22] and then converted to BED files using the bamToBed command of BEDTools (v2.30.0) [23]. Peak calling was performed using model-based analysis of ChIP-seq (MACS) software (v1.4.2) [24]. For the analysis of distribution of m^7^G sites across the mRNA landscape, MetaPlotR was used to calculate and scale the distance of peaks relative to transcriptomic features (https://github.com/olarerin/metaPlotR) [25]. Differentially methylated sites between the two groups were identified with diffReps (v1.55.6) with the criteria of a *P*-value < 0.05 and a fold change (FC) > 2 [26]. Motif analysis was carried out by using discriminative regular expression motif elicitation (DREME) (https://meme-suite.org/meme/tools/dreme). For the input RNA-seq data analysis, raw gene counts were obtained using HTSeq software (v0.9.1) [27], followed by being normalized by the edgeR package (v3.16.5) [28]. Differentially expressed genes (DEGs) were identified according to the following criteria: a fold change > 2 and a *P*-value < 0.05.

DEGs, Differentially methylated genes (DMGs), and common genes between DMGs and DEGs were subjected to Gene Ontology (GO) enrichment analysis using the topGO package (v2.10) [29], and *P*-values were calculated using the hypergeometric distribution method (significant enrichment was defined by a *P*-value < 0.05). For Kyoto Encyclopedia of Genes and Genomes (KEGG) pathway annotation, DEGs, DMGs, and common genes between DMGs and DEGs were searched against the KEGG Database at https://www.genome.jp/kegg/kaas, and enrichment *P*-values were calculated using Fisher’s exact test (significant enrichment was defined by a *P*-value < 0.05).

### Quantitative reverse transcription polymerase chain reaction (RT–qPCR) and methylated RNA immunoprecipitation qPCR (MeRIP–qPCR)

The expression levels of four genes (ETH2_1435900, ETH2_1516900, ETH2_1361700, and ETH2_1248400) were determined by RT–qPCR using the QuantStudio 5 Dx Real-Time PCR System (Thermo Fisher Scientific, Waltham, MA). Actin (ETH2_1310200) was used as the endogenous control [30]. Cycling conditions were as follows: 10 min at 94 °C, followed by 40 cycles of 10 s at 95 °C and 60 s at 60 °C. The comparative cycle threshold (CT) (2^−ΔΔCt^) method was used to calculate the relative RNA expression levels [31]. For validation of the m^7^G sequencing results, both input and m^7^G immunoprecipitation samples were subjected to RT–qPCR analysis. The relative m^7^G enrichment of mRNA was calculated by normalizing to the input: % input = 2^[Ct(input) −Ct(IP)]^ × 1/DF × 100, where DF is the dilution factor between IP and input samples. The sequences of primers used for RT–qPCR and methylated RNA immunoprecipitation–qPCR (MeRIP–qPCR) are shown in Additional file 1: Table S1. Data analysis and statistical tests were carried out using Microsoft Excel and GraphPad Prism 8.0. Numerical data were shown as mean with standard deviation (SD). Differences between the two groups were analyzed by using a Student’s *t*-test. Statistical significance was defined as a *P*-value less than 0.05.

## Results

### General features of m^7^G methylation in unsporulated and sporulated oocysts

**Fig. 1:**
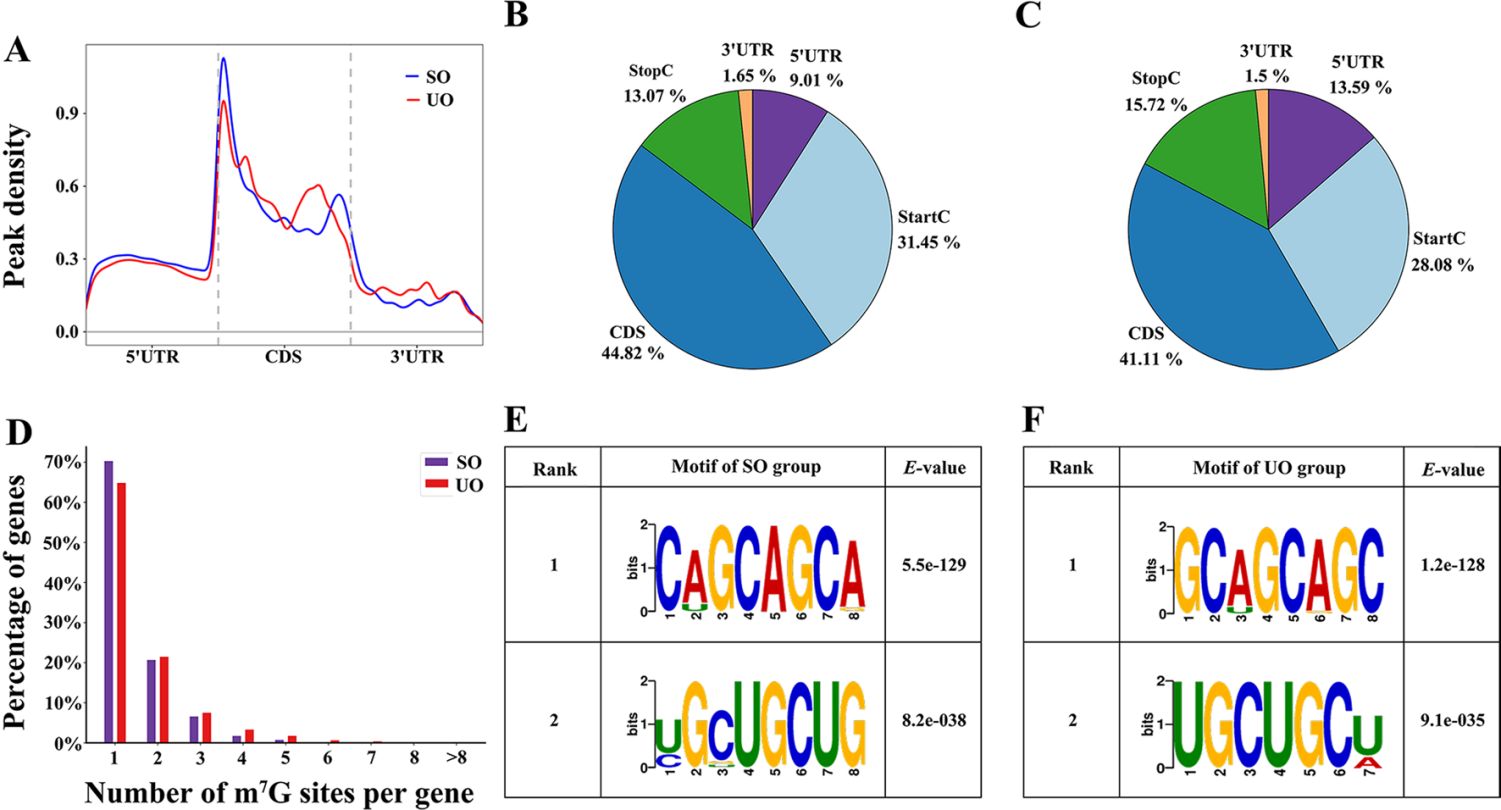
Overview of m^7^G modification in the two groups. **A** Metagene plots displaying the m^7^G peak density distribution across the transcripts. Pie charts exhibiting m^7^G peak distribution in different transcript segments in sporulated (**B**) and unsporulated (**C**) oocysts, respectively. **D** Proportion of genes harboring different numbers of m^7^G peaks in the two groups. **E** The top two sequence motifs enriched from all the identified m^7^G peaks in the SO group. **F** The top two sequence motifs enriched from all the identified m^7^G peaks in the UO group

Through sequencing of methylated RNA immunoprecipitation sequencing (MeRIP-seq) and RNA-seq, 92,388,774–119,399,988 raw reads were generated from each MeRIP-seq dataset and 77,532,536–102,549,048 raw reads were generated from the RNA-seq dataset. After removing low-quality data and successfully mapping to the reference genome, the mean alignment rates were 84.82, 79.56, 77.20, and 75.03% of clean reads for RNA-seq and MeRIP-seq from the unsporulated and sporulated oocysts samples, respectively (Additional file 2: Table S2). We analyzed the distribution patterns of m^7^G peak in the whole transcriptome, and the results showed that m^7^G peaks in each group were highly enriched around start codon region and within CDS region (Fig. 1A). We found that 44.82% of m^7^G peaks were enriched in CDS region, followed by 31.45% in start codon region, 13.07% in stop codon region, 9.01% in 5′UTR region and 1.65% in 3′UTR region in the SO group (Fig. 1B). In the UO group, 41.11% of m^7^G peaks were enriched in CDS region, followed by 28.08% in start codon region, 15.72% in stop codon region, 13.59% in 5′UTR region, and 1.5% in 3′UTR region (Fig. 1C). Subsequently, the number of m^7^G peaks per m^7^G-modified gene was analyzed, and the results showed that more than 80% of genes contained one or two m^7^G peaks in each group (Fig. 1D). In the SO group, ETH2_1256100, a gene encoding a protein containing the kringle domain, harbored the highest number of m^7^G peaks (seven peaks) (Additional file 3: Table S3). In the UO group, ETH2_0101200, a gene encoding a protein containing the N-terminal chorein domain, harbored the highest number of m^7^G peaks (12 peaks) (Additional file 3: Table S3). In addition, motif analysis for the two groups was performed using DREME, and the top two motifs are shown in Fig. 1E, F.

### DMGs and GO enrichment analysis

**Fig. 2:**
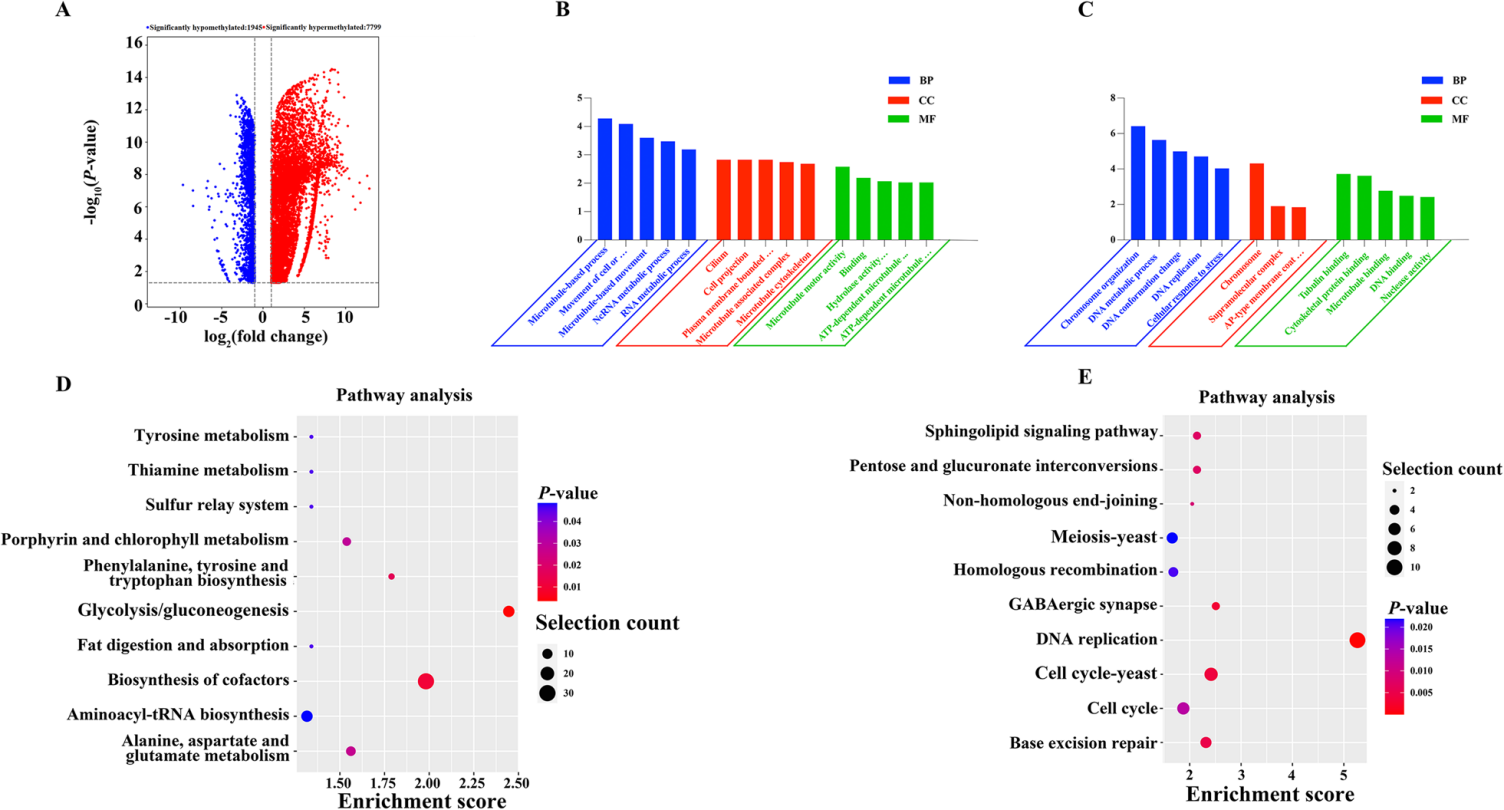
Analysis of differentially m^7^G-modified genes between the two groups. **A** Volcano plots depicting the differential peaks between the studied groups. **B** GO enrichment analysis of the genes with hypermethylated peaks in the SO group. **C** GO enrichment analysis of the genes with hypomethylated peaks in the SO group. **D** KEGG analysis of the genes presenting hypermethylated peaks in the SO group. **E** KEGG analysis of the genes presenting hypomethylated peaks in the SO group

Compared with unsporulated oocysts, 7799 hypermethylated peaks within 3087 genes and 1945 hypomethylated peaks within 847 genes were detected in sporulated oocysts (Fig. 2A, Additional file 4: Table S4). The DMGs were subjected to GO analysis, and the results were grouped into the following three categories: molecular function (MF), cellular component (CC), and biological process (BP). For BP, the genes with hypermethylated peaks were related to microtubule-based process, movement of cell or subcellular component, and microtubule-based movement (Fig. 2B). For CC, the genes with hypermethylated peaks were related to cilium, cell projection, and plasma-membrane-bounded cell projection (Fig. 2B). For MF, the genes with hypermethylated peaks were involved in hydrolase activity, acting on glycosyl bonds, microtubule motor activity, and binding (Fig. 2B).

For BP, the genes with hypomethylated peaks were related to chromosome organization, DNA metabolic process, and DNA conformation change (Fig. 2C). For CC, the genes with hypomethylated peaks were related to chromosome, supramolecular complex, and AP-type membrane coat adapter complex (Fig. 2C). For MF, the genes with hypomethylated peaks were involved in tubulin binding, cytoskeletal protein binding, and microtubule binding (Fig. 2C).

### Pathway enrichment analysis of DMGs

KEGG analysis was performed to further investigate the potential biological function of DMGs. The significantly enriched pathways for the hypermethylated genes included glycolysis/gluconeogenesis, biosynthesis of cofactors, phenylalanine, tyrosine and tryptophan biosynthesis, alanine, aspartate and glutamate metabolism, porphyrin and chlorophyll metabolism, tyrosine metabolism, thiamine metabolism, sulfur relay system, fat digestion and absorption, and aminoacyl-tRNA biosynthesis (Fig. 2D). The significantly enriched pathways for the hypomethylated genes included DNA replication, GABAergic synapse, cell cycle yeast, base excision repair, pentose and glucuronate interconversions, sphingolipid signaling pathway, nonhomologous end joining (NHEJ), cell cycle, homologous recombination, and meiosis yeast (Fig. 2E).

### Integrated analysis of MeRIP-seq and RNA-seq data

**Fig. 3:**
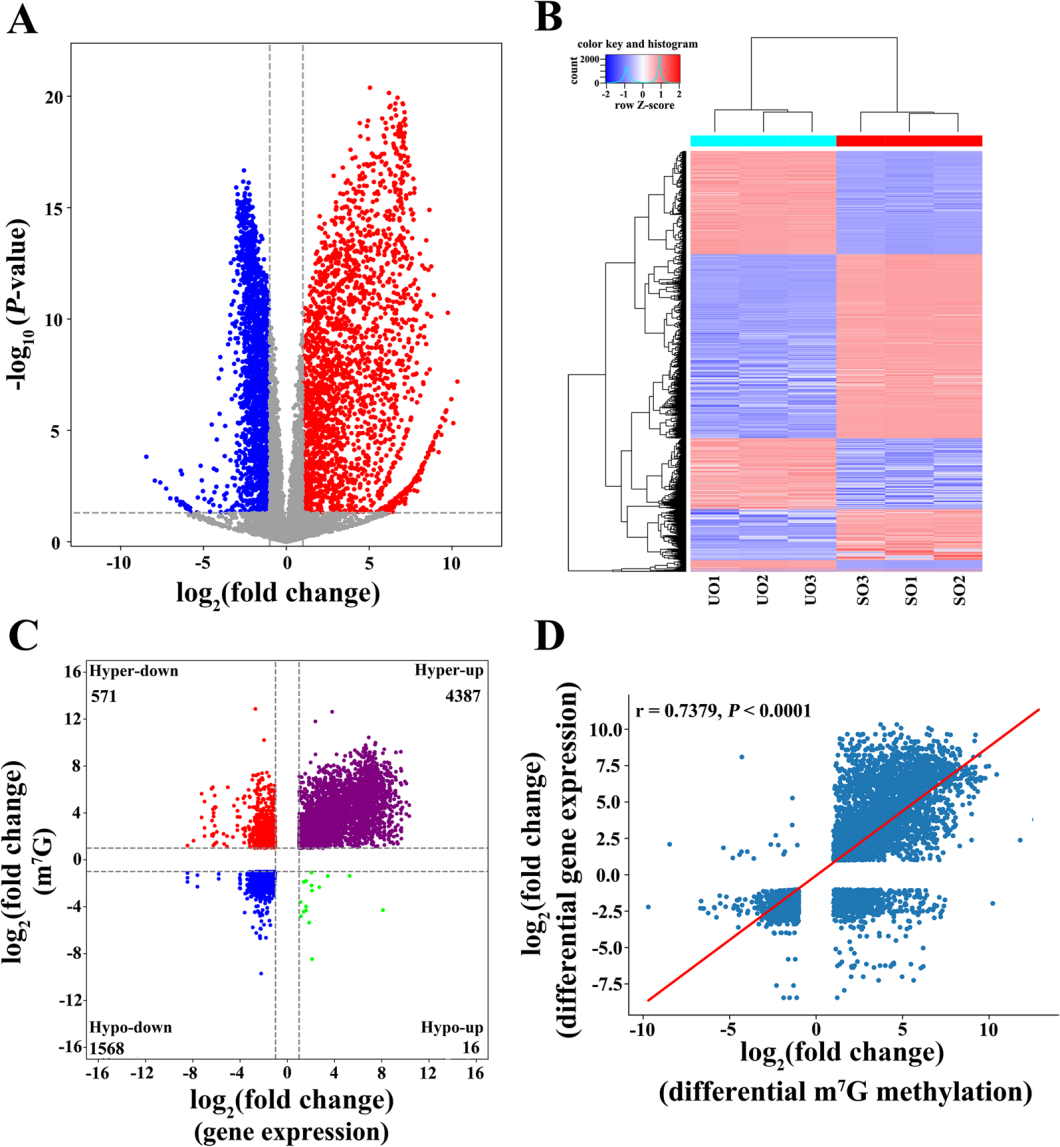
A conjoint analysis of MeRIP-seq and RNA-seq data. **A** Volcano plots of the DEGs between the studied groups. **B** Hierarchically clustered heat maps showing the expression changes between the studied groups. **C** Four quadrant graphs showing the DEGs with differentially methylated m^7^G peaks. **D** Correlation analysis between mRNA expression levels and methylation levels

Compared with the UO group, 4103 genes were differentially expressed in the SO group, including 2306 upregulated genes and 1797 downregulated genes (Fig. 3A, Additional file 5: Table S5). Clustering analysis revealed the distinct gene expression patterns (Fig. 3B). Based on the combined analysis of the RNA-seq and MeRIP-seq data, all DMGs with differential expression were divided into four groups, including 4387 hypermethylated and upregulated (hyper-up) genes, 571 hypermethylated and downregulated (hyper-down) genes, 16 hypomethylated and upregulated (hypo-up) genes, and 1568 hypomethylated and downregulated (hypo-down) genes (Fig. 3C, Additional file 6: Table S6). As shown in Fig. 3D, there was a generally positive correlation between the methylation levels and gene transcript abundance.

**Fig. 4:**
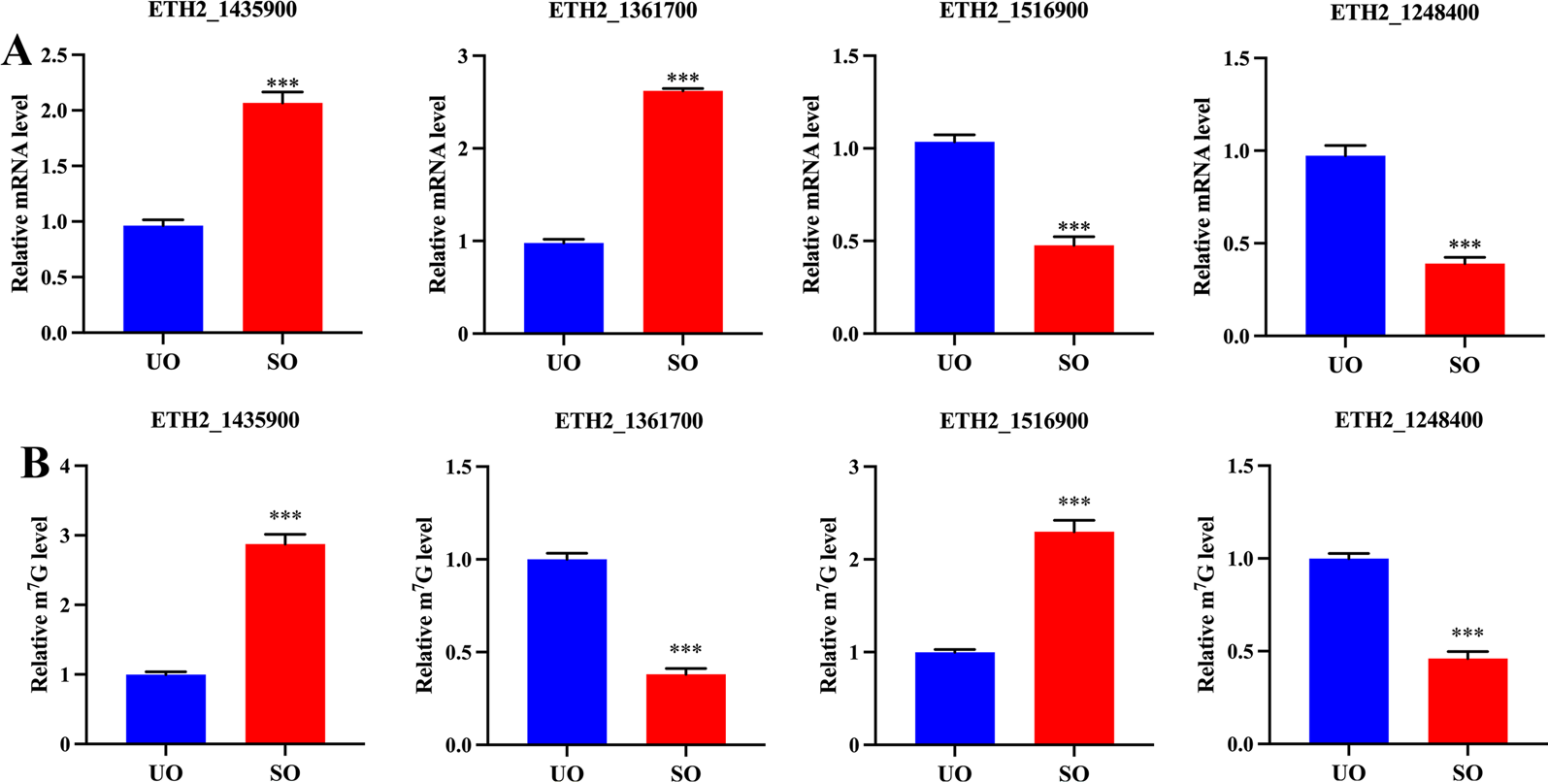
Validation of RNA-seq and MeRIP-seq data. **A** The mRNA levels of four genes were measured by RT–qPCR. **B** The m^7^G levels of four genes were measured by MeRIP–qPCR. **P < 0.01, ***P < 0.001

Four genes (ETH2_1435900, ETH2_1516900, ETH2_1361700, and ETH2_1248400) were subjected to RT–qPCR analysis, and the results were consistent with the gene expression profiles in transcriptome data (Fig. 4A). In addition, a MeRIP–qPCR assay was performed to validate the MeRIP-seq data. The MeRIP–qPCR results were consistent with the sequencing results (Fig. 4B).

**Fig. 5:**
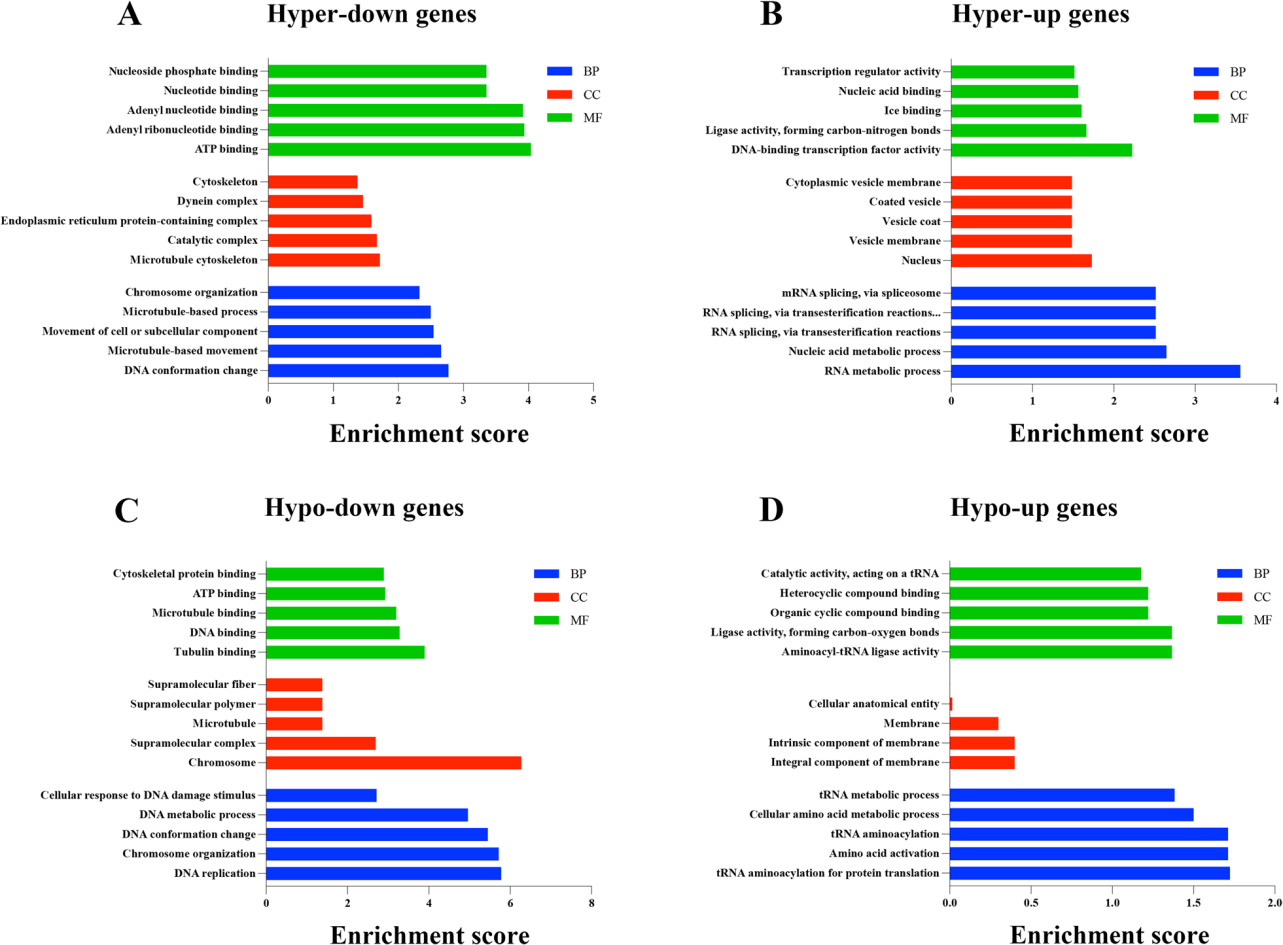
GO analysis of DEGs with altered m^7^G-methylated peaks. **A** Bar plots showing the significantly enriched GO terms for the hyper-down genes. **B** Bar plots showing the significantly enriched GO terms for the hyper-up genes. **C** Bar plots showing the significantly enriched GO terms for the hypo-down genes. **D** Bar plots showing the significantly enriched GO terms for the hypo-up genes

GO analysis showed that the hyper-down genes were mainly associated with DNA conformation change, microtubule cytoskeleton, and ATP binding (Fig. 5A). The hyper-up genes were primarily associated with RNA metabolic process, nucleus, and DNA-binding transcription factor activity (Fig. 5B). The hypo-down genes were involved in DNA replication, chromosome, and tubulin binding (Fig. 5C). The hypo-up genes were mainly associated with tRNA aminoacylation for protein translation, integral component of membrane, and aminoacyl-tRNA ligase activity (Fig. 5D).

**Fig. 6:**
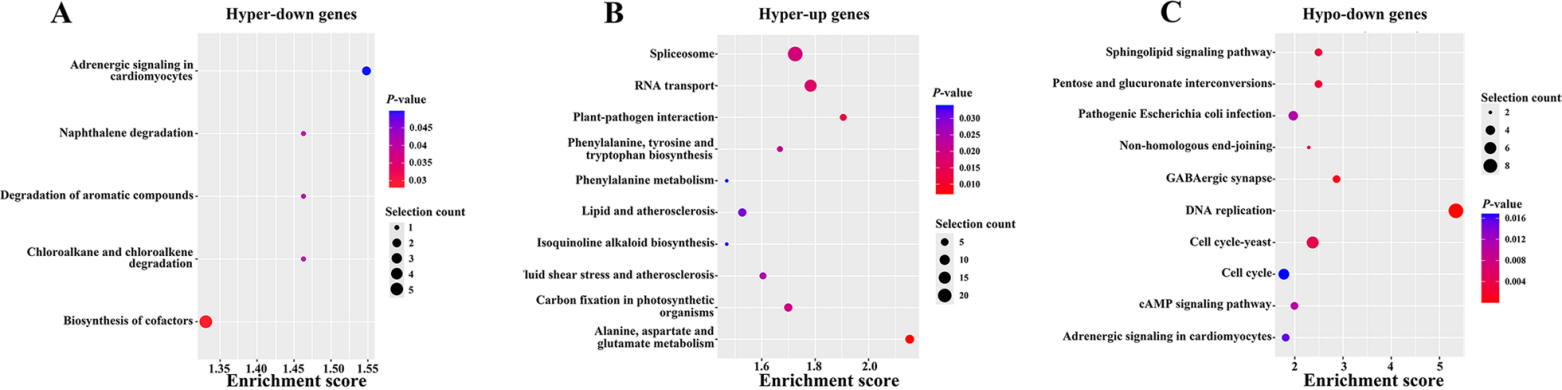
Pathway analysis of DEGs with altered m^7^G-methylated peaks. **A** Bubble charts showing the significantly enriched pathways for the hyper-down genes. **B** Bubble charts showing the significantly enriched pathways for the hyper-up genes. **C** Bubble charts showing the significantly enriched pathways for the hypo-down genes

In addition, KEGG analysis revealed that the hyper-down genes were mainly associated with adrenergic signaling in cardiomyocytes, chloroalkane and chloroalkene degradation, naphthalene degradation, degradation of aromatic compounds, and biosynthesis of cofactors (Fig. 6A). The hyper-up genes were primarily associated with alanine, aspartate and glutamate metabolism, plant-pathogen interaction, RNA transport, spliceosome, carbon fixation in photosynthetic organisms, phenylalanine, tyrosine and tryptophan biosynthesis, fluid shear stress and atherosclerosis, lipid and atherosclerosis, phenylalanine metabolism, isoquinoline alkaloid biosynthesis, tropane, piperidine and pyridine alkaloid biosynthesis, circadian rhythm—plant, morphine addiction, PI3K-Akt signaling pathway, and longevity regulating pathway—multiple species (Fig. 6B). The hypo-down genes were involved in DNA replication, GABAergic synapse, pentose and glucuronate interconversions, sphingolipid signaling pathway, cell cycle-yeast, NHEJ, cAMP signaling pathway, pathogenic *Escherichia coli* infection, adrenergic signaling in cardiomyocytes, cell cycle, parathyroid hormone synthesis, secretion and action, hepatitis B, purine metabolism, autophagy yeast, and meiosis yeast (Fig. 6C).

## Discussion

In the present study, we analyzed, for the first time, the internal mRNA m^7^G modification pattern in unsporulated and sporulated oocysts of *E. tenella*. RT–qPCR and RNA-seq results were in good agreement, demonstrating that the RNA-seq data are reliable. Meanwhile, the reliability of m^7^G-RIP-seq data was validated by MeRIP-PCR. Our findings showed that the overall distribution of m^7^G modification sites is similar in the transcriptome of unsporulated and sporulated oocysts, providing insights into the conservation of m^7^G modification at the two developmental stages. The m^7^G peaks were primarily enriched in the CDS region in each group, indicating that m^7^G modification within the CDS region may play an important role in post-transcriptional gene expression regulation during oocyst development. Intriguingly, a previous study analyzed the distribution patterns of m^7^G peaks across mRNA transcripts in HL60 and HL60/MX2 cells, and the results also showed that the CDS region exhibited the most methylation modification [32]. It was found that the m^6^A peaks were also highly enriched in the CDS region [33]. However, differential distribution patterns were observed. For example, m^6^A enrichment around the stop codon region ranked second [33], whereas m^7^G enrichment around the start codon region ranked second. A start codon is related to translation initiation, while a stop codon ends it [34]. This indicated that m^7^G and m^6^A modifications may play distinct and cooperative roles to ensure accurate mRNA translation initiation and termination during oocyst development, and further studies are needed to better understand the underlying molecular mechanisms. Motif analysis showed that the top two significantly enriched motifs in each group were similar. Strikingly, there are similarities between the motifs identified in the present study and those reported previously in mammalian cells [35].

A large number of DMGs were identified in the SO group compared with the UO group, with hypermethylated genes accounting for the majority. Among them, many genes contained more than one m^7^G peaks. For example, ETH2_0701600 contained two hypermethylated peaks and three hypomethylated peaks. This indicated the involvement of m^7^G modification in post-transcriptional regulation of ETH2_0701600. Multiple processes in *Plasmodium chabaudi* (such as DNA replication, cell cycle, and microtubule-based movements) during the intraerythrocytic developmental cycle were reported to be affected by disruption of serpentine receptor 10 (PcSR10) [36]. Hence, ETH2_0701600, the SR10 homolog in *E. tenella*, may play a regulatory role in the process of sporulation through affecting DNA replication, cell cycle, and microtubule-based movements; if this is the case, besides m^6^A modification [33], m^7^G modification is a component in the complex but well-organized multilayered regulatory network that controls the transcriptome of *E. tenella*. Intriguingly, the DMGs between the two groups were enriched in GO terms of microtubule-based movements; also, significantly enriched KEGG pathways for DMGs included DNA replication and cell cycle.

*E. tenella* genomic DNA double-strand breaks (DSBs) are mainly repaired by NHEJ pathway [37]. In addition, *E. tenella* also relies on homologous recombination for the repair of genomic DSBs [37]. In the present study, both NHEJ and homologous recombination were enriched for the genes with hypomethylated peaks. This indicated the involvement of internal m^7^G methylation in the two pathways for the repair of DSBs in *E. tenella*.

Transcriptome-wide m^6^A profiling of sporulated and unsporulated oocysts of *E. tenella* revealed a positive correlation between m^6^A modification levels of most genes and their mRNA expression levels [33]. Herein, the combined analysis of DMGs and DEGs showed that m^7^G modification levels of the majority of genes was also positively correlated with their mRNA expression levels. Nevertheless, the presence of the hyper-down and hypo-up genes suggested that mRNA m^7^G modification in *E. tenella* may also negatively regulate gene expression.

In the present study, KEGG analysis of the hypo-down genes showed that the significantly enriched pathways included cAMP signaling pathway, autophagy—yeast, and purine metabolism. cAMP signaling pathway and autophagy—yeast were also enriched for the DEGs with altered m^6^A-methylated peaks [33]. This indicated that more than one RNA modifications were involved in the regulation of the same signaling pathway in the stage conversion of *E. tenella*. In addition, purine metabolism is known to provide a cell with the necessary energy [38]. This indicated that m^7^G modification was involved in providing cellular energy. The hyper-up genes were associated with RNA transport; spliceosome; and alanine, aspartate, and glutamate metabolism. Alanine, aspartate, and glutamate are three amino acids that can be de novo synthesized by *Toxoplasma gondii* [39]. It can be inferred that *E. tenella* may possess the ability to synthesize alanine, aspartate, and glutamate, and m^7^G modification was involved in the regulation of amino acid metabolism. In eukaryotes, pre-mRNA splicing catalyzed by the spliceosome is essential for gene expression [40]. Another important step in the control of gene expression is RNA transport [41]. It can be inferred that m^7^G modification occurred in more than one steps of gene expression regulation during oocyst development.

## Conclusions

We present here the first transcriptome-wide map of internal mRNA m^7^G modification in unsporulated and sporulated oocysts of *E. tenella.* We found different methylation features between the two stages and analyzed the potential functions of DMGs and DEGs with altered m^7^G-methylated peaks. The findings provide a solid foundation for further investigation of the molecular mechanisms governing the development of *E. tenella*.

## Supplementary Information

Additional file 1: Table S1. Sequences of primers used for MeRIP–qPCR and RT–qPCR analysis.

Additional file 2: Table S2. A statistical summary of the raw and clean reads.

Additional file 3: Table S3. The number of m^7^G peaks harbored in each methylated gene.

Additional file 4: Table S4. The differentially methylated transcripts between the two groups.

Additional file 5: Table S5. The genes found to be differentially expressed between the two groups.

Additional file 6: Table S6. The genes with significant changes in both m^7^G and mRNA levels.

## Data Availability

The raw sequence data reported in this paper have been deposited in the NCBI Gene Expression Omnibus repository under accession number GSE269982.

## Abbreviations

m^7^G: *N*^7^-methylguanosine
CDS: Coding DNA sequence
DMGs: Differentially methylated genes
DEGs: Differentially expressed genes
mRNA-seq: Messenger RNA sequencing
MeRIP-seq: Methylated RNA immunoprecipitation sequencing
RT–qPCR: Quantitative reverse transcription polymerase chain reaction
GO: Gene Ontology
DSBs: Double-strand breaks
UO: Unsporulated oocysts
SO: Sporulated oocysts
m^6^A: *N*^6^-methyladenosine
m^5^C: methylcytidine
m^1^A: *N*^1^-methyladenosine
mRNAs: Messenger RNAs
rRNAs: Ribosomal RNAs
tRNAs: Transfer RNAs
TAP: Tobacco acid pyrophosphatase
PGM: Porcine gastric mucin-conjugated
RT: Room temperature
MACS: Model-based analysis of ChIP-seq
FC: Fold change
DREME: Discriminative regular expression motif elicitation
KEGG: Kyoto Encyclopedia of Genes and Genomes
SD: Standard deviation
MF: Molecular function
CC: Cellular component
BP: Biological process
NHEJ: Non-homologous end-joining
PcSR10: *Plasmodium chabaudi* serpentine receptor 10

## References

[1] Liu Q, Liu X, Zhao X, Zhu XQ, Suo X. Live attenuated anticoccidial vaccines for chickens. Trends Parasitol. 2023;39:1087–99. 37770352 10.1016/j.pt.2023.09.002

[2] Burrell A, Tomley FM, Vaughan S, Marugan-Hernandez V. Life cycle stages, specific organelles and invasion mechanisms of *Eimeria* species. Parasitology. 2020;147:263–78. 31727204 10.1017/S0031182019001562 PMC10317661

[3] Blake DP, Knox J, Dehaeck B, Huntington B, Rathinam T, Ravipati V, et al. Re-calculating the cost of coccidiosis in chickens. Vet Res. 2020;51:115. 32928271 10.1186/s13567-020-00837-2 PMC7488756

[4] Blake DP, Vrba V, Xia D, Jatau ID, Spiro S, Nolan MJ, et al. Genetic and biological characterisation of three cryptic *Eimeria* operational taxonomic units that infect chickens (*Gallus gallus domesticus*). Int J Parasitol. 2021;51:621–34. 33713650 10.1016/j.ijpara.2020.12.004 PMC8186487

[5] Zaheer T, Abbas RZ, Imran M, Abbas A, Butt A, Aslam S, et al. Vaccines against chicken coccidiosis with particular reference to previous decade: progress, challenges, and opportunities. Parasitol Res. 2022;121:2749–63. 35925452 10.1007/s00436-022-07612-6 PMC9362588

[6] Cowper B, Matthews S, Tomley F. The molecular basis for the distinct host and tissue tropisms of coccidian parasites. Mol Biochem Parasitol. 2012;186:1–10. 22982139 10.1016/j.molbiopara.2012.08.007

[7] López-Osorio S, Chaparro-Gutiérrez JJ, Gómez-Osorio LM. Overview of poultry *Eimeria* life cycle and host–parasite interactions. Front Vet Sci. 2020;7:384. 32714951 10.3389/fvets.2020.00384 PMC7351014

[8] Blake DP. *Eimeria* genomics: where are we now and where are we going? Vet Parasitol. 2015;212:68–74. 25986325 10.1016/j.vetpar.2015.05.007

[9] Chen L, Tang X, Sun P, Hu D, Zhang Y, Wang C, et al. Comparative transcriptome profiling of *Eimeria tenella* in various developmental stages and functional analysis of an ApiAP2 transcription factor exclusively expressed during sporogony. Parasit Vectors. 2023;16:241. 37468981 10.1186/s13071-023-05828-8 PMC10354945

[10] Walker RA, Sharman PA, Miller CM, Lippuner C, Okoniewski M, Eichenberger RM, et al. RNA Seq analysis of the *Eimeria tenella* gametocyte transcriptome reveals clues about the molecular basis for sexual reproduction and oocyst biogenesis. BMC Genomics. 2015;16:94. 25765081 10.1186/s12864-015-1298-6 PMC4345034

[11] Roundtree IA, Evans ME, Pan T, He C. Dynamic RNA modifications in gene expression regulation. Cell. 2017;169:1187–200. 28622506 10.1016/j.cell.2017.05.045 PMC5657247

[12] Frye M, Harada BT, Behm M, He C. RNA modifications modulate gene expression during development. Science. 2018;361:1346–9. 30262497 10.1126/science.aau1646 PMC6436390

[13] Arzumanian VA, Dolgalev GV, Kurbatov IY, Kiseleva OI, Poverennaya EV. Epitranscriptome: review of top 25 most-studied RNA modifications. Int J Mol Sci. 2022;23:13851. 36430347 10.3390/ijms232213851 PMC9695239

[14] Xia X, Wang Y, Zheng JC. Internal m^7^G methylation: a novel epitranscriptomic contributor in brain development and diseases. Mol Ther Nucleic Acids. 2023;31:295–308. 36726408 10.1016/j.omtn.2023.01.003 PMC9883147

[15] Song D, Shyh-Chang N. An RNA methylation code to regulate protein translation and cell fate. Cell Prolif. 2022;55:e13224. 35355346 10.1111/cpr.13224 PMC9136488

[16] Wang C, Hou X, Guan Q, Zhou H, Zhou L, Liu L, et al. RNA modification in cardiovascular disease: implications for therapeutic interventions. Signal Transduct Target Ther. 2023;8:412. 37884527 10.1038/s41392-023-01638-7 PMC10603151

[17] Deng Y, Zhou Z, Ji W, Lin S, Wang M. METTL1-mediated m^7^G methylation maintains pluripotency in human stem cells and limits mesoderm differentiation and vascular development. Stem Cell Res Ther. 2020;11:306. 32698871 10.1186/s13287-020-01814-4 PMC7374972

[18] Fetterer RH, Barfield RC. Characterization of a developmentally regulated oocyst protein from *Eimeria tenella*. J Parasitol. 2003;89:553–64. 12892046 10.1645/GE-3159

[19] Schmatz DM, Crane MS, Murray PK. Purification of *Eimeria* sporozoites by DE-52 anion exchange chromatography. J Protozool. 1984;31:181–3. 6376788 10.1111/j.1550-7408.1984.tb04314.x

[20] Kechin A, Boyarskikh U, Kel A, Filipenko M. cutPrimers: a new tool for accurate cutting of primers from reads of targeted next generation sequencing. J Comput Biol. 2017;24:1138–43. 28715235 10.1089/cmb.2017.0096

[21] Kim D, Langmead B, Salzberg SL. HISAT: a fast spliced aligner with low memory requirements. Nat Methods. 2015;12:357–60. 25751142 10.1038/nmeth.3317 PMC4655817

[22] Li H, Handsaker B, Wysoker A, Fennell T, Ruan J, Homer N, et al. The sequence alignment/map format and SAMtools. Bioinformatics. 2009;25:2078–9. 19505943 10.1093/bioinformatics/btp352 PMC2723002

[23] Quinlan AR, Hall IM. BEDTools: a flexible suite of utilities for comparing genomic features. Bioinformatics. 2010;26:841–2. 20110278 10.1093/bioinformatics/btq033 PMC2832824

[24] Zhang Y, Liu T, Meyer CA, Eeckhoute J, Johnson DS, Bernstein BE, et al. Model-based analysis of ChIP-Seq (MACS). Genome Biol. 2008;9:R137. 18798982 10.1186/gb-2008-9-9-r137 PMC2592715

[25] Olarerin-George AO, Jaffrey SR. MetaPlotR: a Perl/R pipeline for plotting metagenes of nucleotide modifications and other transcriptomic sites. Bioinformatics. 2017;33:1563–4. 28158328 10.1093/bioinformatics/btx002 PMC5860047

[26] Shen L, Shao NY, Liu X, Maze I, Feng J, Nestler EJ. DiffReps: detecting differential chromatin modification sites from ChIP-seq data with biological replicates. PLoS ONE. 2013;8:e65598. 23762400 10.1371/journal.pone.0065598 PMC3677880

[27] Anders S, Pyl PT, Huber W. HTSeq—a Python framework to work with high-throughput sequencing data. Bioinformatics. 2015;31:166–9. 25260700 10.1093/bioinformatics/btu638 PMC4287950

[28] Robinson MD, McCarthy DJ, Smyth GK. edgeR: a Bioconductor package for differential expression analysis of digital gene expression data. Bioinformatics. 2010;26:139–40. 19910308 10.1093/bioinformatics/btp616 PMC2796818

[29] Alexa A, Rahnenführer J, Lengauer T. Improved scoring of functional groups from gene expression data by decorrelating GO graph structure. Bioinformatics. 2006;22:1600–7. 16606683 10.1093/bioinformatics/btl140

[30] Ribeiro E, Silva A, Diallo MA, Sausset A, Robert T, Bach S, et al. Overexpression of *Eimeria tenella* rhoptry kinase 2 induces early production of schizonts. Microbiol Spectr. 2023;11:e0013723. 37260371 10.1128/spectrum.00137-23 PMC10434272

[31] Livak KJ, Schmittgen TD. Analysis of relative gene expression data using real-time quantitative PCR and the 2(−Delta Delta C(T)) method. Methods. 2001;25:402–8. 11846609 10.1006/meth.2001.1262

[32] Zhang B, Li D, Wang R. Transcriptome profiling of *N*^7^-methylguanosine modification of messenger RNA in drug-resistant acute myeloid leukemia. Front Oncol. 2022;12:926296. 35865472 10.3389/fonc.2022.926296 PMC9294171

[33] Liu Q, Mu BJ, Meng YJ, Yu LM, Wang ZR, et al. New insights into developmental biology of *Eimeria tenella* revealed by comparative analysis of mRNA *N*^6^-methyladenosine modification between unsporulated oocysts and sporulated oocysts. J Integr Agric. 2024;23:239–50.

[34] Brito Querido J, Díaz-López I, Ramakrishnan V. The molecular basis of translation initiation and its regulation in eukaryotes. Nat Rev Mol Cell Biol. 2024;25:168–86. 38052923 10.1038/s41580-023-00624-9

[35] Zhang LS, Liu C, Ma H, Dai Q, Sun HL, Luo G, et al. Transcriptome-wide mapping of internal *N*^7^-methylguanosine methylome in mammalian mRNA. Mol Cell. 2019;74:1304-1316.e8. 31031084 10.1016/j.molcel.2019.03.036 PMC6588483

[36] Subudhi AK, O’Donnell AJ, Ramaprasad A, Abkallo HM, Kaushik A, Ansari HR, et al. Malaria parasites regulate intra-erythrocytic development duration via serpentine receptor 10 to coordinate with host rhythms. Nat Commun. 2020;11:2763. 32488076 10.1038/s41467-020-16593-y PMC7265539

[37] Hu D, Tang X, Ben Mamoun C, Wang C, Wang S, Gu X, et al. Efficient single-gene and gene family editing in the apicomplexan parasite *Eimeria tenella* using CRISPR-Cas9. Front Bioeng Biotechnol. 2020;8:128. 32158750 10.3389/fbioe.2020.00128 PMC7052334

[38] Pedley AM, Benkovic SJ. A new view into the regulation of purine metabolism: the purinosome. Trends Biochem Sci. 2017;42:141–54. 28029518 10.1016/j.tibs.2016.09.009 PMC5272809

[39] Krishnan A, Soldati-Favre D. Amino acid metabolism in apicomplexan parasites. Metabolites. 2021;11:61. 33498308 10.3390/metabo11020061 PMC7909243

[40] Yassin SH, Henderson R, Lenberg J, Murillo V, Murdock DR, Friedman J, et al. Further delineation of the CWC27-associated spliceosomeopathy: case report and review of the literature. Am J Med Genet A. 2023;191:1378–83. 36718996 10.1002/ajmg.a.63134

[41] Nakayama K, Kataoka N. Regulation of gene expression under hypoxic conditions. Int J Mol Sci. 2019;20:3278. 31277312 10.3390/ijms20133278 PMC6651685

