# Supplementary material for: Transcriptome-wide mapping of internal mRNA N7-methylguanosine in sporulated and unsporulated oocysts of Eimeria tenella reveals stage-specific signatures
Source: Parasit Vectors. 2024 Nov 27;17:491. doi: 10.1186/s13071-024-06580-3 (PMC11603632; doi:10.1186/s13071-024-06580-3)
Supplement: Supplementary file 1 — Additional file 1: Table S1. Sequences of primers used for MeRIP–qPCR and RT–qPCR analysis. [file 13071_2024_6580_MOESM1_ESM.doc]

**Table S1** Sequences of primers used for MeRIP-qPCR and RT-qPCR analysis

| Locus_tag | Forward primer (5′-3′) | Reverse primer (5′-3′) | Application |
| --- | --- | --- | --- |
| ETH2_1435900 | ATGACCCCAAACTCCAGCAA | AAACGCGGGAACACATGCT | RT-qPCR |
| ETH2_1516900 | GCCGAAAGGGGAAGCAAAC | ACTTTCCTAAGGGCTCACCG | RT-qPCR |
| ETH2_1361700 | TGCTTTCGCCTCCTCTACTT | GTGCAGTTTAAGAACTCACGCA | RT-qPCR |
| ETH2_1248400 | CAGAGGCACAGTCAGCGATA | TGAGCCGTGTTCCTTTGTCC | RT-qPCR |
| ETH2_1310200 (actin) | GAAGAGATGAAGAATGCTGAGG | GATGGATACCCGATGCCTC | RT-qPCR |
| ETH2_1435900 | ATGACCCCAAACTCCAGCAA | AAACGCGGGAACACATGCT | MeRIP-qPCR |
| ETH2_1516900 | GCCGAAAGGGGAAGCAAAC | ACTTTCCTAAGGGCTCACCG | MeRIP-qPCR |
| ETH2_1361700 | TGCTTTCGCCTCCTCTACTT | GTGCAGTTTAAGAACTCACGCA | MeRIP-qPCR |
| ETH2_1248400 | CAGAGGCACAGTCAGCGATA | TGAGCCGTGTTCCTTTGTCC | MeRIP-qPCR |
